# Investigation of Dielectric, Mechanical, and Thermal Properties of Epoxy Composites Embedded with Quartz Fibers

**DOI:** 10.3390/polym15204133

**Published:** 2023-10-18

**Authors:** Imran Haider, Iftikhar Hussain Gul, Muhammad Iftikhar Faraz, Shahid Aziz, Syed Husain Imran Jaffery, Muhammad Ali Khan, Dong-Won Jung

**Affiliations:** 1 Thermal Transport Laboratory, Department of Materials Engineering, School of Chemical & Materials Engineering (SCME), National University of Sciences & Technology (NUST), Islamabad 44000, Pakistaniftikhar.gul@scme.nust.edu.pk (I.H.G.); 2 Department of Mechanical Engineering, College of Engineering, King Faisal University, Al-Ahsa 31982, Saudi Arabia; 3 Department of Mechanical Engineering, Jeju National University, 102 Jejudaehak-ro, Jeju-si 63243, Republic of Korea; 4 Institute of Basic Sciences, Jeju National University, 102 Jejudaehak-ro, Jeju-si 63243, Republic of Korea; 5 School of Mechanical & Manufacturing Engineering (SMME), National University of Sciences & Technology (NUST), Islamabad 44000, Pakistan; 6 Department of Mechanical Engineering, College of Electrical and Mechanical Engineering (CEME), National University of Sciences and Technology (NUST), Islamabad 44000, Pakistan; 7 Faculty of Applied Energy System, Major of Mechanical Engineering, Jeju National University, 102 Jejudaehak-ro, Jeju-si 63243, Republic of Korea

**Keywords:** epoxy composites, dielectric properties, mechanical properties, radome material

## Abstract

Polymer matrix wave transparent composites are used in a variety of high-speed communication applications. One of the applications of these involves making protective enclosures for antennas of microwave towers, air vehicles, weather radars, and underwater communication devices. Material performance, structural, thermal, and mechanical degradation are matters of concern as advanced wireless communication needs robust materials for radomes that can withstand mechanical and thermal stresses. These polymer composite radomes are installed externally on antennas and are exposed directly to ambient as well as severe conditions. In this research, epoxy resin was reinforced with a small amount of quartz fibers to yield an improved composite radome material compared to a pure epoxy composite with better thermal and mechanical properties. FTIR spectra, SEM morphology, dielectric constant (Ɛ_r_) and dielectric loss (δ), thermal degradation (weight loss), and mechanical properties were determined. Compared to pure epoxy, the lowest values of Ɛ_r_ and δ were 3.26 and 0.021 with 30 wt.% quartz fibers in the composite, while 40% less weight loss was observed which shows its better thermal stability. The mechanical characteristics encompassing tensile and bending strength were improved by 42.8% and 48.3%. In high-speed communication applications, compared to a pure epoxy composite, adding only a small quantity of quartz fiber can improve the composite material’s dielectric performance, durability, and thermal and mechanical strength.

## 1. Introduction

Polymer wave transparent composites are used as an integrated component in wireless communication [1]. Polymer composite radomes, which are electromagnetically transparent, are applied to protect fragile antenna systems [2] mounted on different types of aircrafts or aerospace vehicles, radar antennas, and communication and microwave systems from harsh external environments [3]. Their performance depends on how much they affect or interfere with electromagnetic waves or signal transmission [4]. In high-speed communication, low dielectric constant (Ɛ_r_) polymers and their composites are attractive materials for making antenna dielectric layers, printed circuit boards (PCBs), cables, and radomes [4]. Low Ɛ_r_ and low loss factor (δ) are crucial for aircraft radomes, weather radar protection, and other aerospace applications [5]. Reinforcement, matrix, and interface phases make up most composite materials which have benefits of high specific strength, high specific modulus, and great designability [6]. Different manufacturing techniques for reinforced composites include hand lay-up, filament winding, molding, pultrusion, melt mixing, and other molding processes [7].

Wave transmission performance is mostly assessed by the dielectric constant (Ɛ_r_) and dielectric loss factor (δ) of the composite material [8]. The Ɛ_r_ determines the dielectric or polarization of the composite, which is the capacitance ratio of the same size capacitor with dielectrics under vacuum. The δ defines the portion of EM waves that is consumed during its transmission through the material or medium [9]. Relation between the total dielectric loss value (α) of EM wave transmitted in the medium [10] is given by Equation (1):

(1) $$\alpha ={{Ɛ}_{r}}^{1/2}\times f\times tan\delta \;\;\;\;\;\;\;\;\;\;\;\;\;\;\;\;\;\;\;\;$$

where Ɛ_r_ = dielectric constant, *f =* frequency, and *tanδ* = dielectric loss tangent.

**Figure 1:**
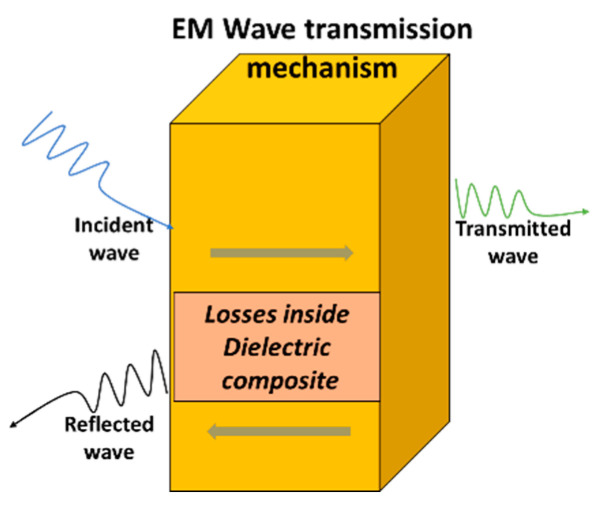
Mechanism of electromagnetic wave transmission.

The mechanism of EM transmission is shown in Figure 1. Dielectric characteristics can significantly and directly impact the performance of electronic devices by resistance–capacitance (RC) delay and crosstalk noise [11]. Stable dielectric materials are characterized by minimal variation in *Ɛ_r_*, a low *δ*, high breakdown strength [12], and mechanical and thermal stability [13]. Equation (2) indicates the relationship between the signal delay time and the losses which is:

(2) $$t_{d}=\frac{l+\sqrt{\varepsilon_{r}}}{c}\;\;\;\;\;\;\;\;\;\;\;\;\;\;\;\;\;\;\;$$

where “*Ɛ_r_*” is the relative permittivity, “*c*” is the speed of light, “*l*” is the transmission distance, and “$t_{d}$” is the signal delay time. Reflection of EM waves mainly occurs at the interface between the medium and air. Impedance matching theory describes that when the *Ɛ_r_* of material is higher, there would be more EM wave reflection, thus reducing the transmission efficiency of electromagnetic waves [14]. The losses occur due to the polarization process of the medium in the external electric field due to EM wave energy loss [15]. Radome materials provide hindrance to electromagnetic waves [16] by reflection and absorption which cause energy loss, resulting in a reduction in communication performance [17].

Furthermore, apart from dielectric properties, many other requirements must be satisfied before the use of polymer materials in high-speed communications [18]. Important requirements include mechanical properties, moisture uptake, adhesion to substrate materials, and chemical and thermal stability [19]. Low Ɛ_r_ polymers that can be used in high-speed communication equipment [18] includes polyimides, bismaleimide, epoxy, phenolic, poly(benzoxazole)s, poly(arylether)s, poly(tetrafluoroethylene), and polyester resin [18] which allow us to make customized shapes of radomes with good performances [19,20]. Epoxy resins are potentially and economically more appealing [20] for preparing low Ɛ_r_ and δ composites [21] as they have the least impact on signal transmission [16]. However, above ambient conditions, their properties can deviate due to their dimensional instability [22] which limits their use in radomes at higher service temperatures [23]. For longer service life applications, there are several challenges for polymeric composite radomes including variations in dielectric properties [10], a lower mechanical integrity, and thermal instability [24]. Besides their dielectric properties, they require reasonable mechanical and thermal strength, durability, a long service life, and low cost [25].

Strategies to mitigate these challenges include replacing these polymer composites with smart materials, ceramics, or metamaterials, using filler doping, surface modification, functionalization, and multilayer dielectric structures, incorporation of porous structure into the polymer to increase air gaps, and use of hybrid composite, nanomaterials, 3D materials, or low-cost alternatives with improved or at least comparable performance. Maintaining long-term and reliable performance at higher temperatures is still a challenge [26]. Some of the scientific and technical challenges include how to (i) realize the synergistic improvement of the wave transmission performance, mechanical properties, and high-temperature resistance through integrated structure/function design for new generation radomes [18]; (ii) design and synthesize high performance composite radomes to withstand high working temperatures while keeping their stability and performance intact [10]; (iii) improve the surface characteristics, interface properties, and interfacial adhesion of fibers; (iv) analyze and characterize the surface performances of fiber composites and the transmission process of electromagnetic wave at the interface; (v) establish wave transparent models to relate the molecular chain electromagnetic wave transmission mechanism and characteristics; and (vi) dynamically simulate the reflection, loss, and transmission process of electromagnetic wave, and to illustrate the transmission mechanism [25]. Glass fibers possess several advantageous characteristics such as low Ɛ_r_ and δ, reasonable mechanical strength, higher softening point, and good adhesion compatibility with epoxy resin which render them suitable as reinforcement in composites. Additionally, they offer benefits, including excellent dielectric properties, high strength to weight ratio, high modulus, corrosion resistance, high-temperature resistance, and a minimal environmental footprint [27]. In a composite structure, the combined area or link between the resin and fibers is known as the interface. The fiber–resin adhesion is responsible for weak or strong interfaces which play an important role in the functionality of reinforced composites [28]. Due to their excellent dielectric, mechanical, and thermal properties, quartz fibers are recognized as the best fibers among the glass fiber family [29]. Moreover, they have good adhesion compatibility with polymeric resins [30].

Compared to pure epoxy composites, reinforced glass fiber composites can withstand higher temperatures (≥300 °C) for longer durations while retaining their mechanical and thermal stability along with the desired electric performance [31]. Glass fibers are suitable in electronic applications and radome manufacturing due to their dielectric stability, low moisture absorption, good mechanical, and thermal stability [32]. However, the final properties of composites depend on the properties and proportion of the constituents, which can be estimated through the rule of mixtures for the evaluation of composite properties [33].

Extensive research has been carried out to investigate the properties of fiber-reinforced composites [12]. Selecting the most suitable reinforcing fibers [34] and polymer matrix [35] is beneficial to obtaining polymer fiber-reinforced composites for radome applications [36]. Keeping in view the above challenges, the current study aimed to fabricate composite reinforcing the polymer matrix with glass fibers for radome applications which will have excellent wave transmission performance, reasonable mechanical strength, and high-temperature resistance. However, this study is concentrated on the influence of embedding quartz fiber quantity to attain combination of specific characteristics include low Ɛ_r_ and low δ, mechanical integrity, and low moisture absorption. A fiber-reinforced composite radome material was fabricated by embedding a small fraction of quartz fibers in epoxy resin. The dielectric constant, dielectric loss, moisture absorption, mechanical properties, and thermal stability were evaluated.

## 2. Materials and Methods

### 2.1. Materials

The experimental materials used in the preparation of reinforced composite samples in this research study were epoxy resin and quartz fibers. Their properties are mentioned in Table 1 and Table 2. Epoxy resin was procured from RESSICHEM Pakistan Ltd. (Karachi, Pakistan). It was a two-part epoxy resin (A) with an amine curing agent (B). Quartz fiber fabric was supplied by Shan Associates Pakistan Ltd. (Lahore, Pakistan)

### 2.2. Composite Property Estimation

Using the rule of mixture (ROM) for evaluating composite properties [39], the dielectric constant and dielectric loss of composite were estimated [40]. The estimated Ɛ_r_ and δ values of the quartz fiber/epoxy composites along with their identification are given in Table 3.

### 2.3. Composite Sample Preparation

**Figure 2:**
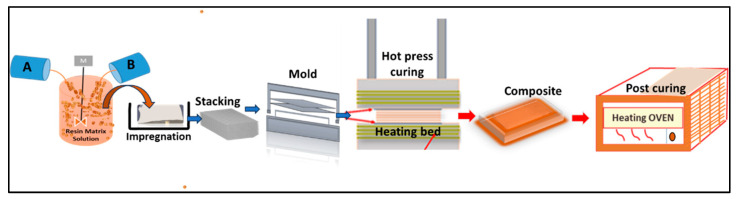
Fabrication of quartz fiber/epoxy composite.

Quartz fiber/epoxy composites were prepared according to the schematic shown in Figure 2. Quartz fiber fabric sheets were weighed, washed with ethanol to remove any organic contamination, and air dried for 30 min. The polymer resin matrix was prepared by adding curing agent (200 gm) slowly to the epoxy resin (500 gm) and then gently mixed. The epoxy resin solution was embedded with 15%, 20%, 25%, 30%, and 35% quartz fabric sheets which were stacked layer by layer in a mold and then hot pressed.

The composite laminates were taken out and then underwent a thermal curing process. The samples prepared for this study were obtained after curing. In Figure.1, “A” refers to epoxy resin, “B” refers to amine curing agent, blue arrow represents cold process (ambient) and red arrow represents hot process (heating).The experimental conditions used for preparation of the quartz fiber/epoxy composites are given in Table 4.

### 2.4. Characterization

The details of the characterization methodologies used to determine the quartz fiber/epoxy composite properties are given in Table 5.

## 3. Results and Discussion

In this section, the experimental results are presented which include the chemical structure, morphology, electrical, mechanical, and thermal properties.

### 3.1. Chemical Structure by FTIR

**Figure 3:**
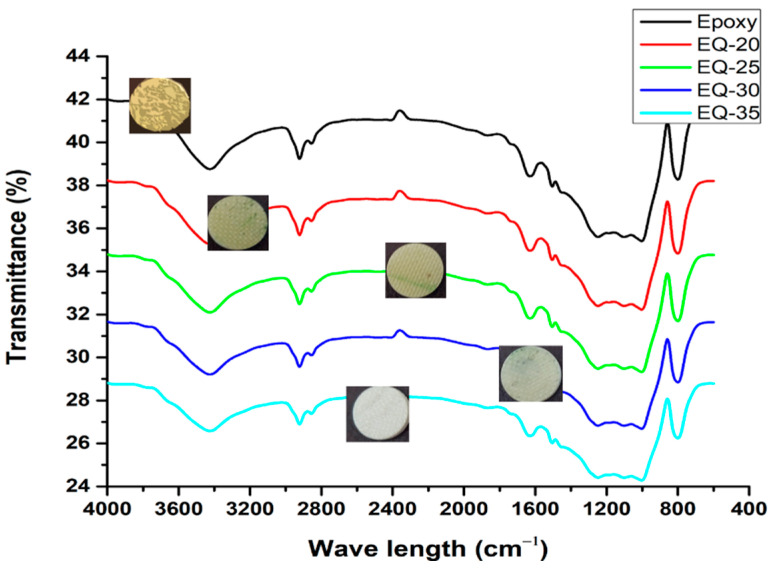
FTIR analysis of quartz fiber (0.20, 0.25, 0.30, 0.35)/epoxy composite.

Figure 3 shows the FTIR spectra of pure epoxy composites and reinforced epoxy composites with an increasing fraction of quartz fibers (0.20, 0.25, 0.3, 0.35). The FTIR spectra showed -OH stretching (bands at 3439 cm^−1^ and 2922 cm^−1^), C-N-C stretching (1174.3 cm^−1^), C=O (1645 cm^−1^), symmetric and asymmetric C-O-C stretching (1099 cm^−1^), C-H deformation (801.2 cm^−1^), Si-O-Si deformation (467.7 cm^−1^), and Si-OH compression (891.3 cm^−1^ and 1005 cm^−1^) bands. The spectra showed polymerization of the thermoset epoxy resin [38] during curing. Irrespective of the embedding quartz fiber content in the epoxy resin, the identical spectra showed the existence of organic and inorganic networks [39] due to the crosslinking of the epoxy/amine upon curing.

### 3.2. SEM Morphology

**Figure 4:**
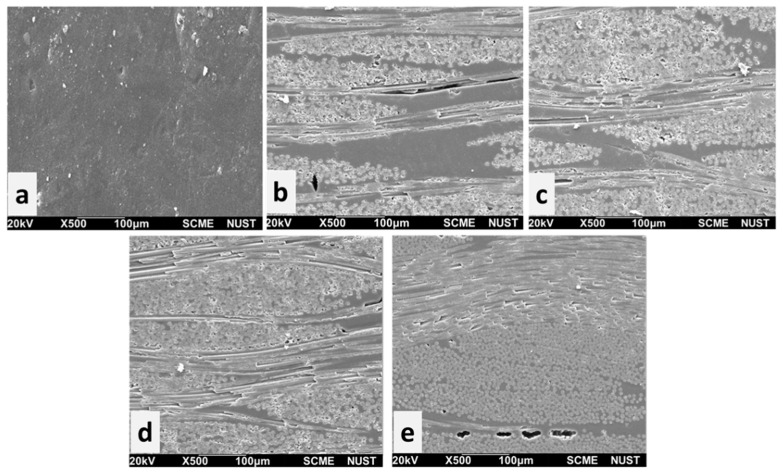
SEM morphology of (**a**) pure epoxy composite, (**b**) 20% quartz fiber, (**c**) 25% quartz fiber, (**d**) 30% quartz fiber, and (**e**) 35% quartz fiber/epoxy composites.

Figure 4a–e shows the SEM morphology of the composites (EQ-20, EQ-25, EQ-30, EQ-35) embedded with increasing fractions of quartz fibers from 0.20 to 0.35. Fiber–resin adhesion can be seen from these images which was due to the fair adhesion compatibility of quartz fibers with epoxy resin. The fair fiber–resin adhesion and increasing quartz fiber proportion showed a better structural appearance of composites. The fibers in EQ-30 and EQ-35 were tightly packed compared to those in EQ-20 and EQ-25 which shows the even distribution with increasing fiber content in the resin. As the fiber fabric fraction increased, the plies were aligned well and tightly bonded and the chance of fiber pull out was reduced. Fewer voids were also observed in the localized areas in these composites which might be due to early crosslinking there or the trapping of air bubbles. Despite the higher fraction of quartz fibers, it might be possible that during layer-by-layer stacking, the fabric plies were moved slightly from their exact location upon pressing. During compaction, the load was transmitted from the liquid resin to the fiber fabric which finally resulted in a uniform and compact composite structure. Overall, the composite structure was improved with the addition of a small amount of quartz fibers.

### 3.3. Electrical Properties

According to the law of energy conservation [21], the electromagnetic (EM) waves passing through a medium are divided into a transmitted wave, reflected wave, and loss [40]. Reflection occurs at the interface between the medium and air [41]. According to impedance matching theory [42], a larger Ɛ_r_ of the material increases the EM wave reflection, with an ultimate reduction in its transmission efficiency due to polarization phenomena of the medium in the external electric field [43].

The dielectric constant (Ɛ_r_) and dielectric loss factor (δ) of the pure epoxy composite was 4.1, and for the quartz fiber/epoxy composite EQ-20, it was 3.31, 3.28 for EQ-25, 3.26 for EQ-30, and 3.26 for EQ-35. These measured Ɛ_r_ and δ values were different from those estimated from the rule of mixture for composite property estimation [44]. The lowest value of Ɛ_r_ was 3.26 with quartz factions of 0.30 and 0.35. The results showed that Ɛ_r_ varied by 1.5% from 3.31. These decreasing values are due to the interfacial polarization of the composite. The dielectric constant became fixed and was not reduced further even with increasing quartz fiber fraction. Increasing the quartz fiber fraction packed the composite structure and reduced the local charge displacement towards the electric field. The epoxy resin embedded with quartz fibers formed a heterogeneous structure due to their organic–inorganic nature. Upon curing of the epoxy resin, space charge polarization was created with the compact interfacial boundaries and random movement of ions was restricted. Thus, ionic conductivity becomes lower with increasing dielectric dispersion in both the real and imaginary parts of Ɛ_r_. In the composites EQ-30 (0.30) and EQ-35 (0.35), due to the relatively lower resin faction compared to EQ-20 and EQ-25, the curing reaction progressed quickly. Thus the epoxy/amine crosslinking contributed towards the dominancy of dipole relaxation over the ionic conductivity, which ultimately reduced the dielectric constant [45]. Moreover, these fiber-reinforced composites are composed of heterogeneous materials that behave differently when exposed to applied electric fields. If they become polarized, their structure leads to polarizability, consequently lowering their Ɛ_r_.

δ of the pure epoxy composite was 0.035, δ of quartz fiber/epoxy composite EQ-20 was 0.030, EQ-25 was 0.028, EQ-30 was 0.021, and that of EQ-35 was 0.021. Increasing the material thickness leads to higher transmission losses, but it’s worth noting that in this study, the thickness remained constant at 3 mm for all composite samples. The presence of quartz fibers changed the composite structure and thus δ was further decreased upon increasing the fiber fraction. Pure epoxy composite exhibits a consistent structure, yet in quartz fiber/epoxy composites, the epoxy, interface, and the fibers collectively contribute to the occurrence of losses. Due to the fast curing, the higher crosslinking density and more stable composite structure resulted in a lower dielectric loss in these composites compared to the pure epoxy/amine composite. Increasing the fiber quantity caused the fibers to embed with epoxy more firmly and restricted the free movement of ions, resulting in a decrease in polarization and reduction in dielectric loss factors by up to 40% from 0.035. The minimum value of δ was 0.021 for EQ-30 and EQ-35. This can be attributed to the fact that the minimum dielectric loss factor did not change even upon an increase in the quartz fraction. It is said that the addition of quartz fiber can significantly reduce the energy dissipation during transmissions of EM waves through this composite material [46]. The performance of wave transparent composites can be estimated by its dielectric loss factor (δ) which is a significant indicator [47]. In high-speed communication, a lower value of δ is critically important as transmission depends directly on the cumulative dielectric loss factors [1,30].

### 3.4. Mechanical Properties

**Figure 5:**
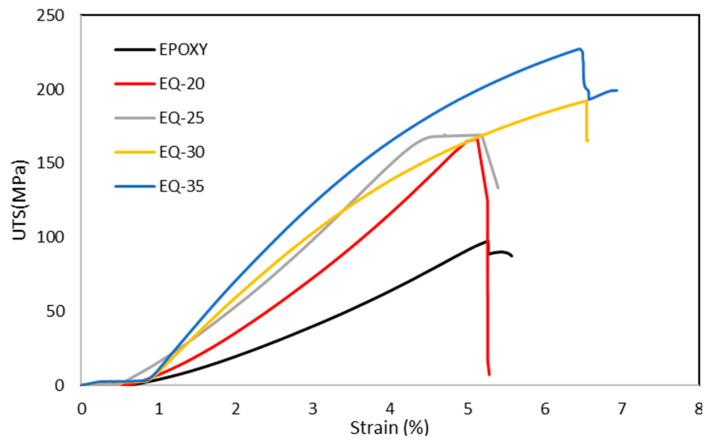
Tensile strength of quartz fiber/epoxy composites.

The tensile strength can be defined as the maximum stress that a material can bear before breaking when it is allowed to be stretched or pulled against the applied load [48]. The tensile strength of the composites in this research were determined and the results are shown in Figure 5. As can be seen from the figure, EQ-35 exhibited the highest tensile strength. In comparison to the pure epoxy composite (97.66 MPa), the addition of a quartz fiber fraction into the composite tended to increase the tensile strength. EQ-20 showed a tensile strength of 166.3 MPa, EQ-25 showed a tensile strength of 170.35 MPa, EQ-30 showed a tensile strength of 194.26 MPa, and EQ-35 showed a value of 226.15 MPa.

**Figure 6:**
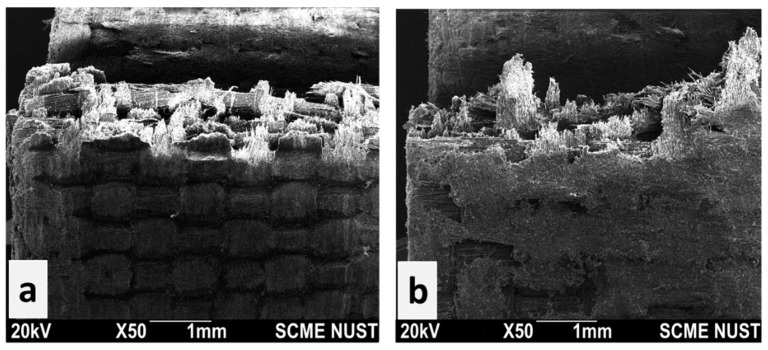
Fractured specimen (35% quartz fibers). (**a**) Tensile test; (**b**) bending strength test.

The results show that the tensile strength improved significantly by adding a small quantity of reinforcing fibers. This increase was mainly due to the presence of quartz fibers. Other contributing factors include fair adhesion of the fiber–resin matrix, crosslinking of the epoxy resin, and applied pressure during composite manufacturing. Due to inclusion of quartz fibers, the composite structure exhibits heterogeneity, which enables it to absorb more stress and exhibits less deformation compared to pure epoxy resin. The fractured specimen is shown in Figure 6; the deformation started from the surface and then a huge amount of energy was absorbed at the fiber–resin interfaces. The tensile load was distributed across the epoxy resin, then at interface and subsequently transferred to the quartz fibers, resulting in overall higher tensile strength.

From these results, we found that tensile strength was enhanced upon increasing the fiber fraction, and EQ-35 (0.35) had the highest tensile strength (226.15 MPa). This was a result of the effective wetting of quartz fibers by the epoxy resin and the improved interfacial bonding resulting from the greater contact area between the fibers and resin. These all contributed to increasing the tensile strength.

**Figure 7:**
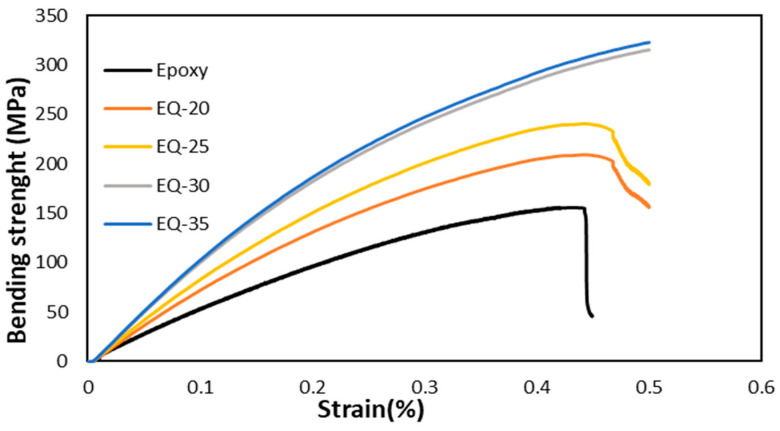
Bending strength of quartz fiber/epoxy composites.

The stress–strain curves obtained during the bend strength test are shown in Figure 7. The bending strength of the pure epoxy composite was 155.3 MPa, that of the quartz fiber/epoxy composite EQ-20 was 209.66 MPa, EQ-25 was 240.63 MPa, EQ-30 was 322.99 MPa, and EQ-35 was 330.69 MPa. Bending strength was found to increase with the increase in quartz fibers due to their better interfacial adhesion with the epoxy resin. The highest value of bending strength was observed for EQ-35 which was 53.03% higher than that of the pure epoxy composite. Embedding quartz fibers enhanced the bending property twofold and the addition of fibers raised the strength in a linear trend. The bending load was distributed among the resin, at the interface, and on reinforcing fibers. The quartz fiber/epoxy composites’ ability to resist and bear bending load was increased. The highest value of bending strength of EQ-35 was 330.69 MPa, which was slightly higher than that of EQ-0.30 (322.99 MPa). The woven quartz fiber fabric evenly absorbed the stresses hence resulted in a higher bending strength. The fractured specimen presented brittle nature of both the quartz fibers and cured epoxy resin as shown by Figure 6. Upon curing epoxies exhibit toughness; nevertheless, the inclusion of quartz fibers imparts a geometric structure and form thereby augmenting their toughness. The increased values of tensile strength and bending strength are indicative of the durable composite structure.

### 3.5. Thermal Degradation (Weight Loss)

**Figure 8:**
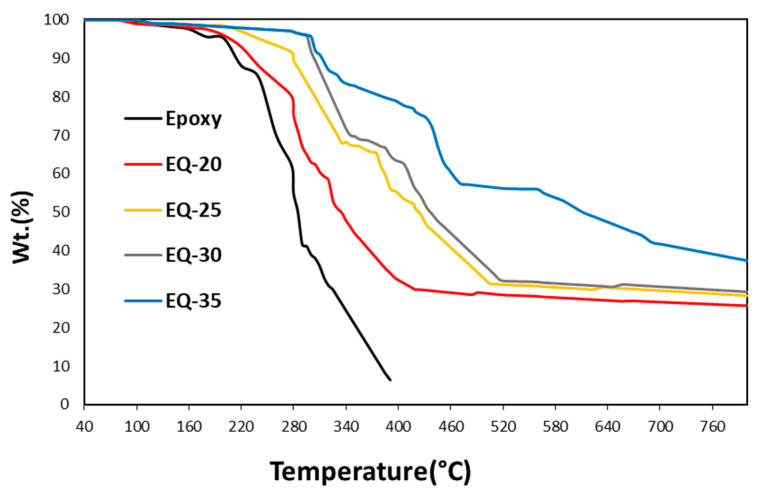
TGA of epoxy and quartz fiber/epoxy composites.

The weight loss (%) of the pure epoxy and reinforced composites is shown in Figure 8. Thermogravimetric analysis (TGA) can determine a material’s thermal stability and thermal decomposition. The weight loss (%) of epoxy, and the EQ-20, EQ-25, EQ-30, and EQ-35 composites were evaluated by TGA with rising temperatures. The initial decomposition of the epoxy composite started from the softening of the polymer composite at 215 °C, followed by a major degradation at 230 °C; then, 70% of the weight was lost at 326 °C, and its further decomposition continued with the rising temperature until its complete degradation.

Apart from the epoxy composite, the quartz fiber/epoxy composites (EQ-20, EQ-25, EQ-30, and EQ-35) decomposed differently with a smaller amount of weight loss. The initial decomposition of sample EQ-20 (0.20 quartz fiber) started at 245 °C; for EQ-25 (0.25 quartz fiber), it started at 280 °C; for sample EQ-30 (0.30 quartz fiber), it started at 290 °C; and for sample EQ-35 (0.35 quartz fiber), it started at 308 °C. Significant degradation in these composites was observed within the temperature range of 285 °C and 650 °C. The major weight loss was observed due to the decomposition of the epoxy resin which is a polymeric component. It was seen that increasing the fraction of quartz fibers provided better thermal stability to the composite compared to the pure epoxy composite. This was evident from the decreasing weight loss of 74.25%, 71.3%, 70.3%, and 64.15%, respectively. Increasing the fraction of quartz fibers reduced the epoxy faction in the reinforced composite.

Moreover, the lower quantity of epoxy resin was efficiently cured by limiting the molecular chain’s thermal vibration up to some extent. In this manner, heat conduction becomes lower and thus, the quartz fiber/epoxy composite exhibited better thermal stability than the pure epoxy composite. Embedding a small quantity of quartz fibers into the epoxy resin increases the manufacturing cost roughly by 15 to 20% when compared to producing pure epoxy composite, all in pursuit of achieving enhanced properties.

## 4. Conclusions

In this work, fiber-reinforced composite radome material was prepared, for communication applications, to withstand high working temperature, mechanical, and thermal stability compared to pure epoxy composites. Our research delved into the impact of embedding quartz fibers and determining their optimal quantity required to achieve enhanced electrical, mechanical, and thermal performance in epoxy composites. Quartz fiber/epoxy (EQ) composites were prepared through hand lay-up, compression molding process followed by thermal curing. FTIR analysis indicated the presence of organic and inorganic networks (-OH, C=O, C-N-C, C-H, and Si-O-Si) while SEM morphology showed fair fiber–resin adhesion, interfacial bonding, and compact structure of EQ composites.

Compared to the pure epoxy resin embedding a small fraction (0.30) of quartz fibers in epoxy, decreased the dielectric constant by 22% (4.10 to 3.21) and the dielectric loss factor by 40% (0.035 to 0.021). This addition of quartz fibers substantially reduced the polarizability of polarized molecules by decreasing their dielectric constant and transmission loss which is advantageous in radome applications. Embedding quartz fiber up to 30% (wt./wt.) significantly improved the mechanical properties with a 49.72% increase in tensile strength and 52.01% rise in bending strength. Beyond this fiber loading, tensile strength was increased but the rise in the bending strength was not exceptional. Moreover, lower weight losses (65%, 44.3%, 39.5%, 36.7%, and 29%) refers to the higher thermal stability of EQ composites when compared to the pure epoxy composite. The enhanced electrical, mechanical, and thermal properties can be attributed to the inclusion of quartz fibers, good interfacial properties, fair adhesion compatibility, and a compact and balanced composite structure. In conclusion, adding a small quantity (0.30) of quartz fibers into an epoxy resin yields excellent dielectric characteristics with improved mechanical and thermal properties. Compared to pure epoxy resin composites, quartz fiber/epoxy composites exhibit substantial potential for applications in radomes.

## Figures and Tables

**Table 1:** Properties of epoxy resin [37].

Phase	Density	Dielectric Constant	Dielectric Loss	Epoxy Content	EEW	Tensile Strength	Tg
	(g/cc)	Ɛ	δ	%	g/g.eq	MPa	°C
Liquid	1.25	3.9–4.3	0.02	23	185	300	215

**Table 2:** Properties of quartz fiber fabric [38].

Weave	Density	SiO_2_	Dielectric Constant	Dielectric Loss	Tensile Strength	Softening Point
Type	(g/cc)	%	Ɛ	δ	GPa	°C
Plain	2.20	99.99	3.78	0.002	1.75	1600

**Table 3:** Identification scheme for quartz fiber/epoxy composites.

Sample ID	EQ-20	EQ-25	EQ-30	EQ-35
Epoxy resin	0.80	0.75	0.70	0.65
Quartz fiber	0.20	0.25	0.30	0.35
Estimated Ɛ_r_	4.03	4.02	4.00	3.98
Estimated δ	0.046	0.045	0.044	0.042

**Table 4:** Preparation conditions for quartz fiber/epoxy composite.

Resin	Fiber	Impregnation	Pressing	Curing Conditions	
Fraction	Fraction	min	bar	Cure	Post Cure
0.80 to 0.65	0.20 to 0.35	30	10	140 °C and 12 h	140 °C & 3 h

**Table 5:** Characterization methodologies for quartz fiber/epoxy composite.

Test	Equipment Used
Functional groups	FTIR- Perkin Elmar Spectrum 100 (Waltham, MA, USA) 400–4000 cm^−1^
Dielectric constant	PNA Network Analyzer 8326 Agilent Frequency used 2 GHz (Santa Clara, CA, USA)
Dielectric loss	
Tensile strength	Trapezium, AGX-Plus, Tokyo, Japan Test speed 2 mm/min, 50 KN
Bending strength	
Interlaminar shear strength	
Morphology	SEM-JSM 6490 A, EOL Tokyo, Japan Accelerating voltage 20 KV
Thermal degradation Weight loss (%)	TGA Q600 SDT, TA Instruments, SHIMADZU, Tokyo, Japan Heating rate = 20° C/min (N_2_ flow)

## Data Availability

Further relevant data can be provided on request.

## References

[1] Clarricoats P.J.B., Rizk M.S.A.S., Parini C.G. (1982). Performance of radome-covered reflector antennas. IEE Proc. H Microw. Opt. Antennas.

[2] Zhou L., Liu Z., Tang L., Pei Y. (2017). Design and characterization for a high-temperature dual-band radome wall structure for airborne applications. Mater. Des..

[3] Munk B.A., Coveyou T.J. (1987). Composite materials: Reactively loaded radomes. Proc. Annu. Southeast. Symp. Syst. Theory.

[4] Zhou X., Liu X., Cui Z., Gu J., Lin S., Zhuang Q. (2020). Design and development of HMS@ZIF-8/fluorinated polybenzoxazole composite films with excellent low-kperformance, mechanical properties and thermal stability. J. Mater. Chem. C Mater..

[5] Nelo M., Liimatainen H., Väätäjä M., Ukkola J., Juuti J., Jantunen H. (2019). Solid air—Low temperature manufacturing of ultra-low permittivity composite materials for future telecommunication systems. Front. Mater..

[6] Lin B., Wang H., Wei J., Sui T. (2021). Diamond wheel grinding characteristics of 3D orthogonal quartz fiber reinforced silica ceramic matrix composite. Chin. J. Aeronaut..

[7] Haris M.Y.M., Aris K.D.M., Zulkifli M., Razak T.A.A., Zuhudi N.Z.M. (2021). Vacuum Infusion Simulation for Radome Manufacturing Using Woven Flax Fibre and Glass Fibre. J. Adv. Res. Fluid. Mech. Therm. Sci..

[8] Othman S., Khalid N.K.A., Seman F.C. (2016). Transmission characteristics of ring periodic array for radome applications. ARPN J. Eng. Appl. Sci..

[9] Chung D.D.L. (2000). Polymer-matrix composites for microelectronics. Polym. Compos..

[10] Tang L., Zhang J., Tang Y., Kong J., Liu T., Gu J. (2021). Polymer matrix wave-transparent composites: A review. J. Mater. Sci. Technol..

[11] Nair R.U., Jha R.M. (2009). Electromagnetic performance analysis of a novel monolithic radome for airborne applications. IEEE Trans. Antennas Propag..

[12] Xue C., Qin Y., Fu H. (2022). Thermal Stability, Mechanical Properties and Ceramization Mechanism of Epoxy Resin/Kaolin/Quartz Fiber Ceramifiable Composites. Polymers.

[13] Zhou L., Pei Y., Fang D. (2016). Dual-Band A-Sandwich Radome Design for Airborne Applications. IEEE Antennas Wirel. Propag. Lett..

[14] Jiang W., Zhang X., Chen D., Ma Y., Yang W. (2021). High performance low-k and wave-transparent cyanate ester resins modified with a novel bismaleimide hollow polymer microsphere. Compos. B Eng..

[15] Jianjun Y., Wensuo M., Zuobin G., Chenhui J., Xianqing L. (2023). Electromagnetic wave-transparent model for 2D woven composites ellipsoid radome. Mech. Adv. Mater. Struct..

[16] Xing Z., Yang F., Yang J., Zhu X. (2023). Low-RCS Ka-band receiving and transmitting satellite communication antennas co-designed with high-performance absorbent frequency-selective radomes. J. Electromagn. Waves Appl..

[17] Agrawal A., Satapathy A. (2017). Mechanical, thermal and dielectric behavior of hybrid filler polypropylene composites. Compos. Commun..

[18] Wang L., Liu C., Shen S., Xu M., Liu X. (2020). Low dielectric constant polymers for high speed communication network. Adv. Ind. Eng. Polym. Res..

[19] Cherukattu Gopinathapanicker J., Inamdar A., Anand A., Joshi M., Kandasubramanian B. (2020). Radar Transparent, Impact-Resistant, and High-Temperature Capable Radome Composites Using Polyetherimide-Toughened Cyanate Ester Resins for High-Speed Aircrafts through Resin Film Infusion. Ind. Eng. Chem. Res..

[20] Choi, Kim J.G., Seo I.S., Lee D.G. (2012). Design of the hybrid composite face with electromagnetic wave transmission characteristics of low-observable radomes. Compos. Struct..

[21] Yuan, Kong X., Wang Q., Wu C. (2020). Intelligent radome design using multilayer metamaterial structures to realize energy isolation and asymmetric propagation of electromagnetic wave. arXiv.

[22] Maier G. (2001). Low dielectric constant polymers for microelectronics. Prog. Polym. Sci..

[23] Polydoropoulou P.V., Katsiropoulos C.V., Pantelakis S.G., Raimondo M., Guadagno L. (2019). A critical assessment of multifunctional polymers with regard to their potential use in structural applications. Compos. B Eng..

[24] Gu J., Dong W., Tang Y., Guo Y., Tang L., Kong J., Tadakamalla S., Wang B., Guo Z. (2017). Ultralow dielectric, fluoride-containing cyanate ester resins with improved mechanical properties and high thermal and dimensional stabilities. J. Mater. Chem. C Mater..

[25] Gu J., Dong W., Xu S., Tang Y., Ye L., Kong J. (2017). Development of wave-transparent, light-weight composites combined with superior dielectric performance and desirable thermal stabilities. Compos. Sci. Technol..

[26] Latrach, Rmili H., Sabatier C., Seguenot E., Toutain S. (2010). Design of a new type of metamaterial radome for low frequencies. Microw. Opt. Technol. Lett..

[27] Wallenberger F.T. (2010). Commercial and experimental glass fibers. Fiberglass and Glass Technology: Energy-Friendly Compositions and Applications.

[28] Elmahdy A., Verleysen P. (2020). Mechanical behavior of basalt and glass textile composites at high strain rates: A comparison. Polym. Test..

[29] Wu Y., Xiao Y., Zou C., Sha X., Gao L., Li S. (2022). High-temperature resistance and wave-transmitting quartz-fibre/polyimide composite. Plast. Rubber Compos..

[30] Bakir M. (2023). Quartz Fiber Radome and Substrate for Aerospace Applications. Eskişehir Tech. Univ. J. Sci. Technol. A Appl. Sci. Eng..

[31] Khajeh A., Mustapha F., Sultan M.T.H., Bánhegyi G., Karácsony Z., Baranyai V. (2015). The Effect of Thermooxidative Aging on the Durability of Glass Fiber-Reinforced Epoxy. Adv. Mater. Sci. Eng..

[32] Wallenberger F.T. (2000). Structural Silicate and Silica Glass Fibers. Advanced Inorganic Fibers: Process-Structure-Properties-Applications.

[33] Shah J.R., Thanki S. (2023). Investigation of the Tensile Properties in Continuous Glass Fiber-Reinforced Thermoplastic Composite Developed Using Fused Filament Fabrication. J. Test. Eval..

[34] Pan L., Ali A., Wang Y., Zheng Z., Lv Y. (2017). Characterization of effects of heat treated anodized film on the properties of hygrothermally aged AA5083-based fiber-metal laminates. Compos. Struct..

[35] Zong L., Zhou S., Sun R., Kempel L.C., Hawley M.C. (2004). Dielectric analysis of a crosslinking epoxy resin at a high microwave frequency. J. Polym. Sci. B Polym. Phys..

[36] Botelho E.C., Nohara E.L., Rezende M.C. (2015). Lightweight structural composites with electromagnetic applications. Multifunctionality of Polymer Composites: Challenges and New Solutions.

[37] Rajamanikandan T., Banumathi S., Karthikeyan B., Palanisamy R., Bajaj M., Zawbaa H.M., Kamel S. (2023). Investigation of dielectric and mechanical properties of Lignocellulosic Rice Husk Fibril for high and medium voltage electrical insulation applications. J. Mater. Res. Technol..

[38] Birsan G., Bria V., Bunea M., Circiumaru A. (2020). An experimental investigation of thermal properties of fabric reinforced epoxy composites. Mater. Plast..

[39] Gonon P., Sylvestre A., Teysseyre J., Prior C. (2001). Combined effects of humidity and thermal stress on the dielectric properties of epoxy-silica composites. Mater. Sci. Eng. B.

[40] Rulf B. (1988). Transmission of microwaves through layered dielectrics—Theory, experiment, and application. Am. J. Phys..

[41] Sebastian M.T. (2008). Dielectric Materials for Wireless Communication.

[42] Joshi S.C., Bhudolia S.K. (2014). Microwave-thermal technique for energy and time efficient curing of carbon fiber reinforced polymer prepreg composites. J. Compos. Mater..

[43] Giere A., Zheng Y., Maune H., Sazegar M., Paul F., Zhou X., Binder J.R., Muller S., Jakoby R. Tunable dielectrics for microwave applications. Proceedings of the 2008 17th IEEE International Symposium on the Applications of Ferroelectrics.

[44] Peters S.T. (1998). Handbook of Composites.

[45] Fujimoto D., Mizuno Y., Takano N., Sase S., Negishi H., Sugimura T. Low-transmission-loss modified cyanate ester materials for high-frequency applications. Proceedings of the 2nd International IEEE Conference on Polymers and Adhesives in Microelectronics and Photonics. POLYTRONIC 2002. Conference Proceedings (Cat. No.02EX599).

[46] Nallayan W.A., Vijayakumar K.R., Rasheed U.T. (2017). Comparison of the Effect of Curing on the Properties of E-Glass/Cyanate modified Epoxy Cross Plied Laminates. IOP Conf. Ser. Mater. Sci. Eng..

[47] Retailleau F., Allheily V., Merlat L., Henry J.F., Randrianalisoa J.H. (2020). Experimental characterization of radiative transfer in semi-transparent composite materials with rough boundaries. J. Quant. Spectrosc. Radiat. Transf..

[48] Pal T., Pramanik S., Verma K.D., Naqvi S.Z., Manna P.K., Kar K.K. (2021). Fly ash-reinforced polypropylene composites. Handb. Fly. Ash.

